# Presenteeism exposures and outcomes amongst hospital doctors and nurses: a systematic review

**DOI:** 10.1186/s12913-018-3789-z

**Published:** 2018-12-19

**Authors:** Juliana Nga Man Lui, Ellie Bostwick Andres, Janice Mary Johnston

**Affiliations:** School of Public Health, Li Ka Shing Faculty of Medicine, The University of Hong Kong, G/F, Patrick Manson Building (North Wing), 7 Sassoon Road, Pokfulam, Hong Kong Special Administrative Region China

**Keywords:** Presenteeism, Work engagement, Burnout, Work demands, Work resources, Productivity, Physicians, Nurses

## Abstract

**Background:**

Presenteeism is a behavior in which an employee is physically present at work with reduced performance due to illness or other reasons. Hospital doctors and nurses are more inclined to exhibit presenteeism than other professional groups, resulting in diminished staff health, reduced team productivity and potentially higher indirect presenteeism-related medical costs than absenteeism. Robust presenteeism intervention programs and productivity costing studies are available in the manufacturing and business sectors but not the healthcare sector.

This systematic review aims to 1) identify instruments measuring presenteeism and its exposures and outcomes; 2) appraise the related workplace theoretical frameworks; and 3) evaluate the association between presenteeism, its exposures and outcomes, and the financial costs of presenteeism as well as interventions designed to alleviate presenteeism amongst hospital doctors and nurses.

**Methods:**

A systematic search was carried out in ten electronic databases from 1998 to 2017 and screened by two reviewers. Quality assessment was carried out using the Critical Appraisal Skills Program (CASP) tool. Publications meeting predefined assessment criteria were selected for data extraction.

**Results:**

A total of 275 unique English publications were identified, 38 were selected for quality assessment, and 24 were retained for data extraction. Seventeen publications reported on presenteeism exposures and outcomes, four on financial costing, one on intervention program and two on economic evaluations. Eight (39%) utilized a theoretical framework, where the Job-Demands Resources (JD-R) framework was the most commonly used model. Most assessed work stressors and resources were positively and negatively associated with presenteeism respectively. Contradictory and limited comparability on findings across studies may be attributed to variability of selected scales for measuring both presenteeism and its exposures/outcomes constructs.

**Conclusion:**

The heterogeneity of published research and limited quality of measurement tools yielded no conclusive evidence on the association of presenteeism with hypothesized exposures, economic costs, or interventions amongst hospital healthcare workers. This review will aid researchers in developing a standardized multi-dimensional presenteeism exposures and productivity instrument to facilitate future cohort studies in search of potential cost-effective work-place intervention targets to reduce healthcare worker presenteeism and maintain a sustainable workforce.

**Electronic supplementary material:**

The online version of this article (10.1186/s12913-018-3789-z) contains supplementary material, which is available to authorized users.

## Background

Presenteeism is a contemporary concept which characterizes the behaviour of employees being physically present at work, but with reduced performance [1]. Presenteeism can be categorized into sickness (physical/ mental) and non-sickness presenteeism (due to personal reasons such as work-life conflict, perceived lack of organizational support, stress etc.) [2].

Presenteeism related exposures differ by sector and are common among staff working in jobs with extensive interpersonal interaction [3]. Extended working time due to globalization, downsizing and individual factors such as gaining facetime at work (a strong feature in the Asian environment) due to job insecurity were found to be major contributors to presenteeism behaviour amongst business, manufacturing and public sector employees [4, 5]. Doctors and nurses faced different pressures contributing to presenteeism, such as difficulties finding a substitute due to manpower shortage and strong organizational culture barriers and professional norms against taking sick leave [6, 7].

Robust presenteeism cross-sector correlational studies [8–10], intervention programs and productivity costing studies have been conducted in the manufacturing and business sectors [11–14] but not the healthcare sector. Employees in the health care (nurses and doctors) and education (full-time school teachers) sectors as compared to 42 occupations across six industries were more prone to presenteeism [10]. In the UK 86% of general practitioners reported presenteeism, followed by > 50% hospital doctors compared to only 32% of office workers [7].

Presenteeism outcomes commonly investigated include frequency of sickness presenteeism [10, 15], presenteeism-related productivity and labor costs [16], and employee health and related medical costs [12, 17]. Presenteeism not only impairs health but also affects team productivity resulting in significant financial costs. The indirect labour costs and medical expenses associated with presenteeism have been estimated to potentially exceed those of absenteeism [18]. As hospital managers seek ways to reduce labor costs which account for more than half of health care institutional expenses while maintaining quality care standards [19], presenteeism productivity costing and intervention studies may provide useful insights regarding targeted interventions or effective methods to improve work performance.

## Aims and objectives

This systematic review aims to 1) identify instruments measuring presenteeism and its exposures and outcomes; 2) appraise the related workplace theoretical frameworks; and 3) evaluate the association between presenteeism, its exposures and outcomes, and the financial costs of presenteeism as well as interventions designed to alleviate presenteeism amongst hospital doctors and nurses.

Review findings will aid the development of a comprehensive scale to identify modifiable intervention targets in reducing nurse presenteeism, useful for hospital managers in formulating evidence-based human resources policies.

## Methods

### Search strategy

Ten electronic databases (Academic Search Premiere, Proquest (British Nursing Index, Medline, PsychINFO), EBSCO (CINAHL,) OVID (Embase (Classic and Global Health)), Pubmed, SCOPUS, and Web of Science) were searched for English peer reviewed publications from 1998 to 2017. For the purpose of this review, the literature search was limited to publications published after 1998, when Simpson defined contemporary presenteeism as “going to work when unfit” [20, 21].

### Screening process

The inclusion and exclusion criteria are described in Table 1. Search terms were selected from the National Library of Medicine’s controlled vocabulary thesaurus – Medical subject headings (MeSH). All ending variations of selected MeSH terms were included by placing the wildcard symbol (*) at the end of the word root. Search terms within themes were incorporated together using the “OR” Boolean operator, while the “AND” Boolean operator was used to combine themes (see Additional file 1). The systematic literature review was conducted following the PRISMA guidelines.

**Table 1:** Inclusion and exclusion criteria

	Inclusion Criteria	Exclusion Criteria
Aims	Primarily examine the exposures, institutional outcomes and impacts of presenteeism or sickness attendance of hospital frontline employees (nurses and doctors)	Publications that examine population-wide findings, burden of disease and patient-related
Setting	Hospital in-patient (primary, secondary and tertiary settings)	Outpatient, rehabilitation and nursing homes
Population	Full time frontline healthcare workers (doctors and nurses).	Administrative-related staff, residents, student trainees and other healthcare professionals (e.g. radiographers, laboratory technicians)
Design	Cross-sectional or cohort research design randomized controlled trials	Qualitative, questionnaire validation studies
Modifiers	Organizational and individual psychosocial exposures of presenteeism outcomes of presenteeism financial costs of presenteeism intervention programs on presenteeism	Non-modifiable personal traits (e.g. personality), population disease burden

### Publication selection

Publication titles and abstracts were screened and for abstracts that failed to provide satisfactory support for the inclusion/exclusion criteria, the full text was reviewed. Two reviewers independently assessed the quality and accuracy of the selected publications and one reviewer screened the publications and tabulated the relevant information. Any discrepancies between the two reviewers were resolved through discussion until consensus was reached. Reference lists of selected publications and those published ahead of print were also manually screened.

### Quality assessment

The Critical Appraisal Skills Program (CASP) tool was adopted to assess the quality of selected publications. The CASP tool includes unique checklists for evaluating eight different types of studies. In this systematic review, the CASP cohort study checklist (modified for both cohort and cross-sectional studies) and the economic evaluation checklist were adopted [22]. The two checklists each contain 12 dichotomous response questions and two questions summarizing study results. Items 6a and 6b of the cohort study tool (length and completeness of follow up) were excluded when assessing cross-sectional studies for a possible total score of 12 for cohort and economic studies and 10 for cross sectional studies. Studies that scored six or above for prospective/ cohort studies and five or above for economic evaluation studies were selected for data extraction.

### Data extraction

Data extraction by study type tabulated presenteeism related 1) exposures and outcomes, 2) financial costs, and 3) intervention outcomes. Extracted elements included the following:- a) author, publication year and country, b) sample characteristics (population and response rate), c) survey method, d) type of presenteeism studied (sickness, non-sickness, overall), recall period and measuring, e) financial costing method or intervention (for financial costing or intervention studies only), f) measurement instruments and their validity, and g) results of the study.

Quality of presenteeism exposures and outcomes measures in assessed studies were evaluated based on its scale selection and reliability, scales with satisfactory Cronbach’s alpha values > 0.7 will be considered for multidimensional scale development in our next stage.

Categorization of presenteeism and its exposures and outcomes were primarily based on the most commonly used framework in our assessed studies, the JD-R model. Work-related factors were categorized into four domains: work stressors (job aspects that require substantive mental and physical effort), work resources (job aspects that stimulate personal development, help achieve work goals), work psychosocial emotions (employee emotional and mental outcomes from work) and work outcomes (work performance indicators). Individual factors were categorized into three domains: demographics, individual health (mental, psychological and physical health) and personal factors (factors outside of work).

## Results

**Fig. 1:**
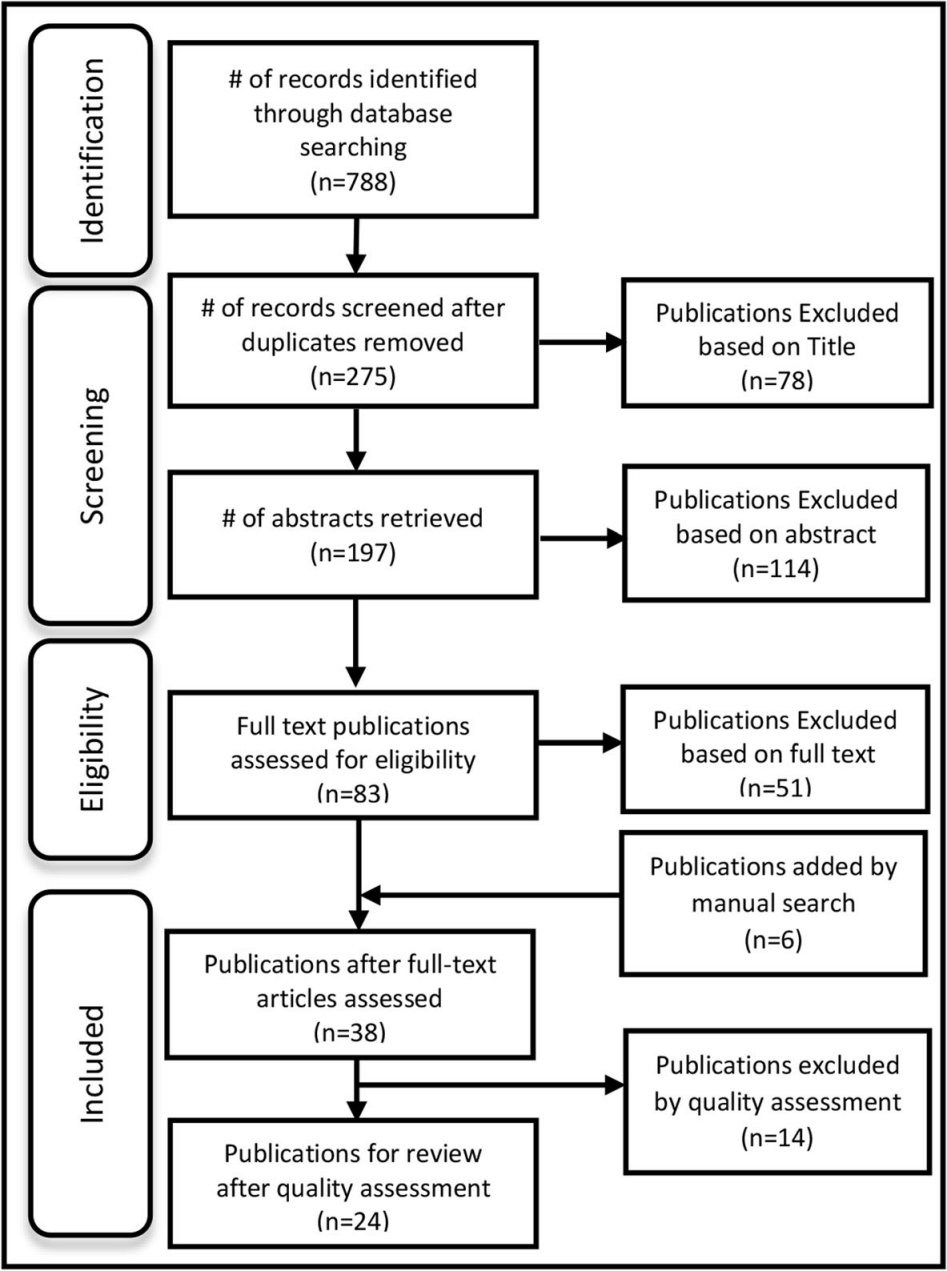
Flow diagram of literature search results

In the initial search, 788 peer-reviewed publications were identified. After removing duplicates, 275 unique publications remained. One hundred and ninety-two publications were screened out based on title and /or abstract leaving 83 publications for full text screening. Of these, 32 were selected for inclusion. Six additional publications were identified by manual search for a total of 38 publications for quality assessment (see Fig. 1).

Of the 38 publications, 61% (*N* = 22/36) passed the CASP-cohort quality assessment tool, and 100% (*N* = 2/2) passed the CASP-economic evaluation tool, leaving 24 publications for final inclusion in the systematic review (see Additional file 2).

Presenteeism exposures and outcomes were reported in 17 publications, in which three (18%) used a prospective study design [23–25], and 14 (82%) a cross sectional study design [4, 7, 26–36]. All four presenteeism financial costing publications used a cross-sectional study design [18, 37–39]. Of the three randomized controlled trials on the effectiveness of presenteeism intervention programs [40], two were economic evaluations [41, 42].

Twenty (83%) of the publications included in the systematic review were published after 2010. Twenty-two (92%) drew from Western populations (Europe [4, 23, 24, 27, 28, 30–33, 36, 38–42], North America [18, 25, 26, 29, 37], United Kingdom [7]) and only two (8%) from non-western locales (South America [34] and China [35]). Ten (42%) sampled only nurses [18, 23, 25, 27–29, 34, 41, 42], four (17%) only doctors [4, 30–32] and ten (42%) a variety of health care professionals [7, 24, 26, 33, 35–40].

Nine (38%) used a paper-based survey with response rates of 47–86% [4, 7, 18, 23–26, 29, 39], four (17%) used a web-based survey with response rates of 26–86% [30, 33, 36, 37], two (8%) used a mixture of web and paper survey with response rates approximating 53% [31, 32], and the remaining did not specify the survey method but reported response rates of 49–91% [27, 28, 35, 38, 40–42].

While all exposure and financial costing studies had clearly stated aims, only 16 (73%) adopted an appropriate sampling framework, recruitment strategy or response rate (anonymized, randomized, and > 50% response rate) (Additional file 2). Sixteen (73%) measured presenteeism exposures and 13 (59%) measured outcomes using valid and reliable tools. Ninenteen (86%) considered confounding factors. Of the prospective studies, only two followed up with subjects appropriately [24, 25]. Data extraction on study characteristics and results of selected publications are listed in Additional file 3.

### Presenteeism exposures and outcomes measures

Quality assessment on presenteeism organizational and individual exposures and outcomes measures are presented in Tables 2 and 3. Presenteeism exposures and outcomes were measured either by adopting validated scales or self-derived items across assessed studies. Most work-related adopted scales reported satisfactory reliability with Cronbach’s alpha > 0.7.

**Table 2:** Presenteeism work-related exposures and outcomes measures identified in reviewed publications

Organizational Exposures/ outcomes measured	Adopted scale origins	Publications	Reliability (Cronbach’s α)
Work stressors			
Time pressure	Effort-Reward Imbalance scale	*Dellve 2011*	0.57–0.78
Job demands/ work pressure	Furda 1995	*Demerouti 2009*	0.83–0.85
Physical demands	Self-derived items	*Demerouti 2009*	–
Ergonomic factors (stooping)	Dutch Musculoskeletal Questionnaire	*d’Errico 2013*	0.64–0.86
Patient demands	Herschbach 1992	*Demerouti 2009*	0.80–0.85
Musculoskeletal pain/disease	Self-derived items	*Dellve 2011*	–
	World Health Organization’s Health and Work Performance Questionnaire (HPQ)	*Warren 2011*	–
	Self-derived items	*Letvak 2012*	0.72–0.99
	Self-derived items	*Martinez 2012*	–
	Nordic questionnaire of musculoskeletal symptoms	*Trinkoff 2006*	–
Role conflicts	Self-derived items	*Sendén 2013*	–
Organizational justice	Moorman 1991	*d’Errico 2013*	0.70
Working group climate	Francis and Young 1979	*d’Errico 2013*	0.88
Quality of working process	Francis and Young 1979	*d’Errico 2013*	0.77
High Responsibility	Effort-Reward Imbalance scale	*Dellve 2011*	0.57–0.78
Limited lifting equipment	Nordic Musculoskeletal Disorder Questionnaire	*Skela-Savič 2017*	–
Work resources			
Effort-reward balance	Effort-Reward Imbalance scale	*Dellve 2011*	0.57–0.78
Decision making/ work pace control	General Nordic Questionnaire for Psychological & Social Factors at Work (QPS Nordic)	*Thun 2014*	0.45–0.84
Income	Self-derived items	*Martinez 2012*	–
Institutional flu measures	Self-derived items	*LaVela 2007*	–
Organizational care	The General Nordic Questionnaire for Psychological and Social Factors at Work	*Sendén 2013*	role conflict: 0.74, organizational care: 0.83
Social support	Self-derived item	*Dellve 2011*	–
	Self-derived item	*Thun 2014*	–
Supervisory support	McAllister 1995	*d’Errico 2013*	0.89
	Andersen 2010	*Thun 2014*	–
Affective commitment	Mowday 1979	*Yang 2017*	0.85
Work Psychosocial Emotions			
Optimism/ positive work feelings/ daily activity well-being/ meaningfulness	Self-derived items	*Dellve 2011*	–
	Hospital Anxiety & Depression scale (single positive item)		–
Job satisfaction	Self-derived items	*Rantanen 2011*	–
Burnout	Maslach Burnout Inventory Dutch version	*Demerouti 2009*	depersonalization: 0.62–0.68, emotional exhaustion: 0.86–0.90
	Shirom-Melamed Burnout Questionnaire	*Dellve 2011*	0.97
	Maslach Burnout Inventory (MBI)	*d’Errico 2013*	0.71–0.91
	Oldenburg Burnout Inventory	*Thun 2014*	exhaustion: 0.80, disengagement 0.77
	Utrecht Burn-out Scale	*Vandenbroeck 2017*	–
Psychological stress/	Nordic Questionnaire for Psychological & Social Factors at Work (single item)	*Dellve 2011*	–
	Self-derived items	*Martinez 2012*	–
	Challenge and hindrance-related self-reported stress (C-HSS)	*Yang 2017*	0.87–0.75
	Inventory of stress in nurses (ISN)	*Umann 2014*	interpersonal relationships: 0.90, roles of stressors: 0.82, work intrinsic factors: 0.79
Work dissatisfaction	Perceived Stress Scale (PSS 10)	*Skela-Savič 2017*	0.86
Work Outcomes			
Medication errors	Self-derived item	*Letvak 2013*	–
Patient falls	Self-derived item	*Letvak 2013*	–
Quality of care	Kramer 2004	*Letvak 2013*	0.80–0.90

**Table 3:** Presenteeism individual exposures and outcomes measures identified in reviewed publications

Organizational Exposures/ outcomes measured	Adopted scale origins	Publications	Reliability (Cronbach’s α)
Work-related Characteristics			
Age	Self-derived item	*Letvak 2013*	–
		*Martinez 2012*	–
		*Sendén 2013*	–
		*d’Errico 2013*	–
		*Heponiemi 2013*	–
		*Thun 2014*	–
		*Aysun 2017*	–
Hospital/ Department	Self-derived item	*d’Errico 2013*	–
Employer	Self-derived item	*Heponiemi 2013*	–
Being in academia	Self-derived item	*Sendén 2013*	–
Marriage status	Self-derived item	*d’Errico 2013*	–
Country	Self-derived item	*Sendén 2013*	–
Ethnicity	Self-derived item	*Warren 2011*	–
Job title	Self-derived item	*d’Errico 2013*	–
		*Aysun 2017*	–
		*Warren 2011*	–
Seniority/ work experience	Self-derived item	*Martinez 2012*	–
		*d’Errico 2013*	–
Rank	Self-derived item	*Skela-Savič 2017*	–
Shift work	Self-derived item	*Rantanen 2011*	–
Gender	Self-derived item	*Martinez 2012*	–
		*Sendén 2013*	–
		*Thun 2014*	–
		*Senden 2016*	–
		*Aysun 2017*	–
		*Demerouti 2009*	–
Sick leave policy	Self-derived item	*Sendén 2013*	–
Type of employment (perm)	Self-derived item	*d’Errico 2013*	–
Work schedule (full-time)	Self-derived item	*d’Errico 2013*	–
Sick leave	Self-derived item	*Sendén 2013*	–
		*Dellve 2011*	–
Hours worked	Self-derived items	*Martinez 2012*	–
Individual health			
Acute/chronic disease	Self-derived items	*Rantanen 2011*	–
	WHO-HPQ	*Warren 2011*	–
Overall health symptoms	Self-derived items	*Martinez 2012*	–
Mental symptoms/ conditions (depression/ anxiety)	Wellness-at-Work Survey	*Warren 2011*	–
	Patient Health Questionnaire (PHQ-9)	Letvak 2012	0.89
	Self-derived item	Martinez 2012	–
	Avallone 2005	d’Errico 2013	–
Lower back pain interference with daily activities	Nordic Musculoskeletal Disorder Questionnaire	Skela-Savič 2017	–
	Dutch Musculoskeletal Questionnaire	d’Errico 2013	0.72
Medication/ vaccination	Self-derived items	*LaVela 2007*	–
Dutifulness	Self-derived item	Dellve 2011	–
Good general health	Johansson and Lundberg 1996	*Demerouti 2009*	–
	Eriksson 2004	*Dellve 2011*	–
	Self-derived items	*Martinez 2012*	–
	Short-form health survey derived item	*Aysun 2017*	–
Self-diagnosis and treatment	Self-derived items	*Sendén 2013*	–
Need for healthcare provision/ pharmacotherapy	Nordic Musculoskeletal Disorder Questionnaire	Skela-Savič 2017	–
	Wellness-at-Work Survey	*Warren 2011*	–
Sleep problems	Wellness-at-Work Survey	*Warren 2011*	–
Decreased physical activities	Nordic Musculoskeletal Disorder Questionnaire	Skela-Savič 2017	–
Poor Work Ability	Work Ability Index	*Dellve 2011*	–
Decreased Performance	Hagberg 2002	*Dellve 2011*	–
Personal factors			
Coping strategies	Occupational Coping scale (ECO)	*Umann 2014*	control: 0.77–0.81
Social support at home	Self-derived item	Dellve 2011	–
Work-family/ Family work conflict	Self-derived item	*Dellve 2011*	–
	Netemeyer 1996	*d’Errico 2013*	0.77–0.86
	Derived from QPS Nordic	*Senden 2016*	–
	Self-derived items and van Exel 2004	Boumans 2014	–

Many of the adopted presenteeism exposures and outcomes scales were culturally-specific and focused on a Western work environment, for example the Dutch musculoskeletal questionnaire, Nordic questionnaire of musculoskeletal symptoms, and general Nordic questionnaire for psychological and social factors at work.

Commonly studied exposures/ outcomes included musculoskeletal pain/disease [18, 24, 25, 27, 37], burnout [23, 24, 28, 32, 36], psychological stress [7, 24, 34, 35], age [4, 27–29, 31, 32, 38], gender [23, 27, 30–32, 38], mental symptoms/ conditions [18, 27, 37] and general health [23, 24, 27, 38]. Despite the common exposures and outcomes investigated, scales adopted across studies vary.

### Presenteeism measures

Four publications reported a dichotomous measure of presenteeism experience (yes/no) [4, 7, 25, 26], seven measured presenteeism frequency on a three to seven-point Likert scale [23, 24, 30–33, 36] and 12 measured presenteeism-related productivity/ work lost using validated Likert-scale composite scores, or hours or days of work lost [18, 27, 29, 34, 35, 37–42] (Table 4). Validated composite Likert-scales measuring presenteeism-related productivity include the Work Productivity and Activity Impairment (WPAI) scale, Stanford Presenteeism Scale (SPS-6) and Work Limitations Questionnaire (WLQ), Perceived Ability to Work Scale (PAWs) and the World Health Organization Health and Performance Questionnaire (HPQ).

**Table 4:** Presenteeism measures identified in reviewed publications

Presenteeism measure	Adopted scale origins	Response options	Recall period	Publications
Sickness presenteeism frequency	Single item (Aronsson 2000)	4-point Likert scale	1 year	*Demerouti 2009*
				*Dellve 2011*
		3-point Likert scale		*Boumans 2014*
	Physician Career path questionnaire (PCPQ)	5-point Likert scale	Non-specified	*Sendén 2013*
	Self-derived item (Senden 2013)	5-point Likert scale	Non-specified	*Thun 2014*
	Self-derived items	5-point Likert scale	1 year and lifelong	*Senden 2016*
	Self-derived item	5-point Likert scale	6 months	*Vandenbroeck 2017*
Sickness presenteeism experience	Derived from Standard Shiftwork Index	Dichotomous (yes/no)	6 months	*Trinkoff 2006*
	Self-derived items	Dichotomous (yes/no)	Non-specified	*Mckevit 1997*
	Self-derived items	Dichotomous (yes/no)	6 months	*LaVela 2007*
	Self-derived items	subjects with lower back pain (LBP) reporting no days of absence for LBP	1 year	*d’Errico 2013*
	Self-derived item	Dichotomous (yes/no)	1 year	*Heponiemi 2013*
Sickness presenteeism productivity	World Health Organization health and work performance questionnaire (HPQ)	0–10 score	1 week	*Warren (2011)*
	1 item from Work productivity and activity impairment questionnaire (WPAI)	0–10 score		
	Stanford Presenteeism Scale (SPS-6) (α =0.78–0.82)	5-point Likert scale	1 month	*Martinez 2012*
	WPAI	0–10 score	2 weeks	*Letvak 2012*
				*Letvak 2013*
	Work limitations questionnaire (WLQ) (α = 0.8–1.0)	0–100 score	2 weeks	*Umann 2014*
	SPS-6 (α = 0.67)	5-point Likert scale	1 month	*Skela-Savič 2017*
	Perceived ability to work scale (PAWS) (α = 0.89)	0–10 score	Not specified	*Yang 2017*
	Derived from HPQ (top 20 health problems in business sector)	Number of work days/hours	2 weeks	*Aysun (2017)*
	Self-derived item	0–100 score on 10 cm visual analogue scale	1 month	*Rantanen (2011)*
	HPQ	0–10 score	1 month	*Christensen 2015*
	Nurses Work Functioning Questionnaire (NWFQ) and Productivity and Disease Questionnaire (PRODISQ)	7-point Likert scale	Not specified	*Noben (2014)*
		0–1 efficiency score on 10-point scale days worked	6 months	
	PRODISQ	0–1 efficiency score on 10-point scale days worked	6 months	*Noben (2015)*

While the presenteeism recall periods varied from 7 days to 1 year, publications reporting productivity loss/costs had shorter recall periods (7 days-1 month) [18, 27, 29, 34, 37–40], publications reporting on presenteeism experience or frequency had longer periods (6 months-1 year) [4, 23–26, 28, 30, 33, 36, 41], and five did not specify a recall period [7, 31, 32, 35, 41].

### Theoretical frameworks

Eight (33%) utilized a theoretical framework to guide the study analysis, whereas five (21%) used an individual (Sickness Flexibility Model, Health-driven Economic Burden Model, Role-stress Theory, Effort-recovery Model, Theory of Stress and Coping and Challenge Stressor-Hindrance Stressor Framework) based framework [24, 33–35, 37] and three (13%) used a framework that combined organizational and individual factors (Job Demands-Resources (J-DR) model, Dynamic Model of Presenteeism and Absenteeism, Demands Control model and Effort-reward Imbalance model) [23, 27, 36] (see Table 5). The most commonly used framework was the J-DR framework model [23, 36].

**Table 5:** Theoretical Frameworks in selected papers

Individual Frameworks	Publications	Work Psychosocial frameworks	Publications
Sickness Flexibility Model (Johansson 2007)	*Dellve 2011*	Derived from Job Demands-Resources (JD-R) model (Bakker 2000)	Demerouti 2009
Presenteeism as Health-Driven Economic Burden Model (McGinni 2002)	*Warren 2011*	The dynamic model of presenteeism and absenteeism (Johns 2010)	Martinez 2012
Role-stress theory (Kahn et al. 1964) Effort–recovery (E-R) model	*Boumans 2014*	JD-R model	Vandenbroeck 2017
Theory of Stress and Coping (Lazarus)	*Umann 2014*		
Derived from challenge stressor-hindrance stressor framework (Lepine 2005, Podsakoff 2007)	*Yang 2017*		

### Association between presenteeism and related exposures and outcomes

The association between presenteeism and its related exposures and outcomes in assessed studies were primarily categorized on the most commonly used framework JD-R model in Tables 6 and 7.

**Table 6:** Association between presenteeism and work-related exposures and outcomes

Organizational-related Factors	Exposures			Outcomes		
	Positive	Negative	Not significant	Positive	Negative	Not significant
Work stressors						
Time pressure	Dellve 2011					
Job demands/ work pressure	Demerouti 2009					
Physical demands	Demerouti 2009					
Ergonomic factors (stooping long)		d’Errico 2013				
Patient demands	Demerouti 2009					
Musculoskeletal pain/disease	Dellve 2011			Trinkoff 2006		
	Warren 2011					
	Letvak 2012					
	Martinez 2012					
Role conflicts	Sendén 2013					
Organizational justice	d’Errico 2013					
Working group climate	d’Errico 2013					
Quality of working process			d’Errico 2013			
High Responsibility	Dellve 2011					
Limited lifting equipment	Skela-Savič 2017					
Work resources						
Effort-reward balance, Reward		Dellve 2011				
Decision making/ work pace control		Thun 2014				
Income		Martinez 2012				
Institutional flu measures		LaVela 2007				
Organizational care		Sendén 2013				
Social support		Dellve 2011				
		Thun 2014				
Supervisory support		Thun 2014	d’Errico 2013			
Affective commitment		Yang 2017				
Work Psychosocial Emotions						
Optimism/ positive work feelings/ daily activity well-being/ meaningfulness		Dellve 2011				
Job satisfaction		Rantanen 2011				
Burnout (Overall)				Dellve 2011		
Burnout (Exhaustion)	Demerouti 2009		d’Errico 2013	Demerouti 2009		
	Vandenbroeck 2017			Thun 2014		
Burnout (Depersonalization/ disengagement)			d’Errico 2013	Demerouti 2009		
			Vandenbroeck 2017	Thun 2014		
Burnout (Personal Competence)	Vandenbroeck 2017					
Psychological stress	Dellve 2011					
	Martinez 2012					
	Umann 2014					
	Yang 2017					
Work dissatisfaction	Skela-Savič 2017					
Work Outcomes						
Medication errors				Letvak 2013		
Patient falls				Letvak 2013		
Quality of care				Letvak 2013

**Table 7:** Association between presenteeism and individual exposures and outcomes

Organizational-related Factors	Exposures			Outcomes		
	Positive	Negative	Not significant	Positive	Negative	Not significant
Demographics						
Age	Letvak 2013	Aysun 2017	Sendén 2013			
	Martinez 2012		d’Errico 2013			
			Heponiemi 2013			
			Thun 2014			
Hospital/ Department			d’Errico 2013			
Employer (Public sector)	Heponiemi 2013					
Being in academia	Sendén 2013					
Marriage status			d’Errico 2013			
Country	Sendén 2013^i^					
Ethnicity			Warren 2011			
Job title (nurses)	d’Errico 2013		Warren 2011			
	Aysun 2017					
Seniority/ work experience		Martinez 2012	d’Errico 2013			
Rank (non-managerial)	Skela-Savič 2017					
Shift work			Rantanen 2011			
Gender (female)	Martinez 2012	Demerouti 2009				
	Sendén 2013					
	Thun 2014					
	Senden 2016					
	Aysun 2017					
Compensatory leave/ sick leave policy	Sendén 2013					
Type of employment (perm)			d’Errico 2013			
Work schedule (full-time)			d’Errico 2013			
Sick leave	Sendén 2013			Dellve 2011		
Hours worked	Martinez 2012					
Individual health						
Acute/chronic disease	Warren 2011					
	Rantanen 2011					
Overall health symptoms	Martinez 2012					
Mental symptoms/ conditions (depression/ anxiety)	Warren 2011		d’Errico 2013			
	Letvak 2012					
	Martinez 2012					
Lower back pain interference with daily activities			d’Errico 2013	Skela-Savič 2017		
Influenza medications/ vaccine-related behaviours			LaVela 2007			
Dutifulness	Dellve 2011					
Poor general health	Demerouti 2009			Dellve 2011		
	Martinez 2012					
	Aysun 2017					
Self-diagnosis and treatment	Sendén 2013					
Need for healthcare provision/ pharmacotherapy	Skela-Savič 2017					
	Warren 2011					
Sleep problems	Warren 2011					
Decreased physical activities	Skela-Savič 2017					
Poor Work Ability				Dellve 2011		
Decreased Performance				Dellve 2011		
Personal factors						
Coping strategies			Umann 2014			
Social support at home		Dellve 2011				
Work-family/ Family work conflict	Dellve 2011		d’Errico 2013			
	Senden 2016					
	Boumans 2014					

Presenteeism was positively associated with most work stressors [18, 23, 24, 27, 28, 31, 37], but not extensive stooping and working process quality [28]. All tested work resources were negatively associated with presenteeism [24, 26, 27, 31, 32, 35], except for the contradictory findings between presenteeism and supervisory support [28, 32].

Work psychosocial factors such as job satisfaction and other positive work emotions were negatively associated with presenteeism [24, 39]. d’Errico, Demerouti and Vandenbroeck presented contrasting evidence on the relationship between exhaustion and burnout and presenteeism [23, 28, 36]. Others found the depersonalization subdomain of burnout was not significantly associated with presenteeism [28, 36].

Contradictory results were noted in the associations between age [4, 27–29, 31, 32, 38], profession (i.e., nurse) [28, 37, 38], seniority/ work experience [27, 28] and presenteeism. Presenteeism was found to be positively associated with working in the public sector [4], academia [31], non-managerial grades [43], having a paid leave policy [31], number of days of sick leave [31] and hours worked [27]. However, presenteeism had no significant association with the workplace setting [28], marital status [28], ethnicity [37], shift-work schedule [39], permanent employment contracts [28] or full-time employment [28].

Employee health problems were studied in relation to presenteeism as both exposures and outcomes. Presenteeism was positively related to most employee health exposures such as acute/chronic diseases, overall health symptoms and poor general health [23, 27, 37–39]. Discrepancies exist in the findings on associations between lower back pain interference with daily activities [28, 43], mental symptoms/ conditions (depression/anxiety) and presenteeism [18, 27, 28, 37]. All health outcomes were positively associated with presenteeism, such as decreased physical activities, sleep problems, poor work ability and decreased performance [24]. As for non-health related personal exposures, most studies investigated work-family conflict with all but one study finding a positive association with presenteeism [24, 28, 30, 33].

### Financial costing, intervention and economic evaluation studies

The Human Capital Costing Method (HCM) was used in all financial costing and economic evaluation studies [37, 38, 41, 42] except for one which used a contingent valuation method [39]. Sickness presenteeism productivity costs ranged from USD $2000 – $15,541 per healthcare employee annually [18, 37–39]. Sickness presenteeism costs (USD $340 /person) were lower than sickness absenteeism costs (USD $463 /person) [39]. All financial costing methods considered overall sickness presenteeism productivity costs. Productivity costs in Letvak’s study were for nurses only, while other publications considered productivity costing for a mixture of occupations (e.g. doctors, pharmacists, dentists, administrators etc.).

A randomized controlled trial (diet, physical activity, and cognitive behavioural training intervention) had limited short term effect in reducing mental health related presenteeism whereas an occupational physician follow-up intervention in two different hospitals was found to be cost effective in reducing mental health related presenteeism in two economic evaluation studies [41, 42]. An e-mental health program was not found to be cost-effective (ICER: − 0.047) compared to the occupational physician intervention (ICER: 0.033) [41].

## Discussion

This systematic review is the first to extensively examine multi-dimensional presenteeism organizational exposures measures, productivity financial costing, intervention studies and related psychosocial frameworks within the hospital-based healthcare workforce context. Most of the reviewed publications were cross-sectional in nature and few reported financial costing, economic evaluation or interventions. The contradictory associations found between presenteeism and common exposures or outcomes across studies limit decisive conclusions for the health care field. Although the concept of “presenteeism” first appeared in late twentieth century [44], there is no agreement on its precise operational definition.

While self-derived single-item measures may be suitable for numerical or categorical answers (e.g. number of medication errors, patient falls, demographics), though often criticized when measuring complex psychometric constructs (e.g. social support, mental conditions) due to low content validity (difficulty in representing a complex theoretical concept), limited sensitivity (more items provide more interval points on scale for discrimination) and restricted reliability evaluation (at least a two-item scale needed in evaluating consistency) [45].

Cross-cultural adaptation studies may also be needed before applying scales developed for a specific population to a different cultural context. Moreover, some commonly investigated exposures/ outcomes (e.g. burnout, psychological stress and general health) were measured by varying adopted scales across studies limiting comparability, as constructs may be conceptualized differently across scales. For example, different conceptualized burnout measures were compared amongst nurses and a two-factor structure (exhaustion and withdrawal) was confirmed to be most favourable. Development of a standardized multi-dimensional presenteeism exposures and outcomes scale may alleviate limitations on comparability for commonly investigated factors of interest. Adopted scales with satisfactory reliability (Cronbach’s alpha > 0.7) in the assessed studies will be considered for our scale development in the next stage.

Most commonly researched exposures and outcomes focus on medical factors, such as stress, burnout, general health, psychological symptoms and conditions, mostly due to the abundance of occupational health studies. The evidence presented here focuses on the impact of work demands and negative work psychosocial emotions (e.g. burnout, stress and dissatisfaction) as compared to work resources and positive work psychosocial emotions (e.g. satisfaction, positive work feelings, meaningfulness). Existing research on presenteeism exposures amongst nurses focuses on the effect of job design on psychosocial emotions and work productivity. The relationship between organizational policies, leadership style and organizational culture on presenteeism have been widely researched in other sectors but not among doctors and nurses [44]. For a more multi-dimensional and comprehensive overview on presenteeism exposures and productivity effects amongst nurses, future research should expand on these potentially impactful but rarely researched exposures.

In this review, presenteeism is measured either by experience, frequency and/or productivity. Operationally some researchers adopted a dichotomized response set to ask whether participants ever experienced presenteeism (yes or no) [21–23] and thereby limited the utility of the presenteeism data [4, 7, 25, 26]. The Likert scales adopted by others [30–32] with inherent numerical assumptions limit outcome comparability. Others have used self-reported workplace presenteeism productivity measurement instruments with variable outcome metrics and others still have used presenteeism productivity outcomes which focus on specific disease states rather than overall health-related productivity [44, 46]. Additionally, in most cases a more nuanced analysis of the impact of seasonality, work related factors and stressors was limited.

There is wide variation in presenteeism recall periods amongst selected studies. In a multi-sector productivity audit in the United States, Stewart studied variation in presenteeism recall periods of one, two, and four weeks [47]. The most accurate recall period for health-problem related presenteeism is 2 weeks. Elsewhere researchers used longer recall periods to capture frequency and experience of presenteeism episodes [47]. Variation in recall periods leads to questions about the accuracy of self-reported presenteeism, which has not been considered in most of the studies in this review, a problem which has been highlighted in other reviews [47–50]. With few objective presenteeism and presenteeism productivity loss measures, the accuracy of self-reported presenteeism and absenteeism is difficult to establish. There are however, well established methods for verifying self-report scale precision, such as using retrospective diary data [51, 52]. Progress on the standardization and validation of presenteeism metrics and its monetary conversion methods has been stagnant since Schultz’s systematic review on employee health and presenteeism [53]. Contradictory and limited comparability on findings across studies may be attributed to variability of selected scales for measuring both presenteeism and its exposures/outcomes constructs.

### Financial costs of presenteeism

Financial costing and economic evaluations valued productivity using the HCM in all but one of the selected publications. HCM often provides the most conservative and highest estimates compared to other costing methods, such as contingent valuation method (CVM) and friction cost method (FCM). HCM is a better estimation method in cost of illness studies, as it avoids self-selection bias possible with CVM when participants respond to hypothetical willingness-to-pay scenario questions, and is comparatively easier for researchers to implement [54].

### Presenteeism intervention and economic evaluation studies

There is a paucity of intervention and economic evaluation studies amongst hospital doctors and nurses. The research here focuses on mental health improvements whereas others have investigated the effectiveness of workplace health intervention delivery methods across occupational sectors [11] or specific interventions such as back pain improvement, lighting changes, extra rest break time, telephone support and occupational health [11]. The lack of standardized multi-dimensional presenteeism exposures and productivity measures based on sound theoretical frameworks contributes to the scarcity of intervention and economic evaluation research amongst healthcare workers.

### Use of theoretical frameworks to guide research

While much of the included research was not guided by a theoretical framework, some used psychosocial frameworks at both the individual and organizational levels. These considered the interaction between organizational work factors and individual psychosocial emotions. Individual psychosocial frameworks are more appropriate for occupational health related research that aims to improve employee health from an individual perspective.

Existing work psychosocial frameworks include 1) JD-R model, 2) job-demands control (JD-C) model and 3) effort reward imbalance (ERI) model. JD-C model hypothesizes that job stress level depends on the interaction between job demands and individual decision latitude on job control. ERI model hypothesizes that stress arises when received rewards at work are not in line with the perceived effort put in by employees. Both the JD-C and ERI model constrain organizational research to a limited number of negative exposures [55]. The JD-R model considers the dual effect of work resources (positive) and work demands (negative) on work psychosocial emotions [56].

Presenteeism research to date has been limited by theocratical frameworks mostly focusing on medical or individual health related research. However, organizational exposures (e.g. leadership style and organizational culture) were shown to impact presenteeism behaviour and work performance [57, 58]. The authors of JD-R model have recently proposed an updated model to consider multi-level (organizational, team) effects on individual employee presenteeism [59]. This could greatly assist hospital managers in formulating evidence-based human resources policies in the future.

### Potential cross-cultural differences in presenteeism behaviour

The included studies were predominantly conducted in Western jurisdictions. However, for the one study conducted in Asia, stress levels and presenteeism behaviour were significantly higher amongst Chinese employees as compared to their British counterparts [60]. These cross-cultural differences may be explained by the underlying Chinese traditional values of Confucianism and collectivism which emphasize endurance and hardwork [60].

## Limitations

Methodological limitations in our study include but are not limited to the following. Firstly, meta-analysis was not undertaken for the selected publications due to the heterogeneity of presenteeism outcome measures and limited number of studies. Secondly, limiting searches to only English language publications may have restricted the inclusion of publications written in Asian languages such as Korean, Japanese and Chinese. Thirdly, with the potential measurement error and conceptual differences between presenteeism instruments, comparison of presenteeism association with related exposures and economic costs between studies must be interpreted with caution. Lastly, limited cohort, intervention and economic valuation publications were available, confining the generalizability and heterogeneity of results.

## Conclusion

In this systematic review, no conclusive evidence can be drawn on the association between presenteeism and its exposures amongst hospital healthcare workers based on the heterogeneity and limited quality of measurement tools. More evidence is needed to confirm the relationship between presenteeism positive exposures and outcomes (e.g. job satisfaction, social and supervisory support) amongst healthcare employees, and their feasibility as intervention targets. Based on our findings, researchers should consider theoretical frameworks with multi-level interaction which would allow for vertical and horizontal comparisons within and between organizations (e.g. leadership style and organizational culture) and individual exposures or outcomes in the future [59]. The limited number of economic evaluation studies with non-standardized instruments and varying costing methods restrict the estimation and comparison of presenteeism productivity costs amongst healthcare workers across studies. A standardized multi-dimensional presenteeism exposures and productivity instrument should be developed to facilitate cohort studies from both East and West in search of potential cost-effective and cultural-specific work-place intervention targets to reduce healthcare worker presenteeism and maintain a sustainable workforce.

## Additional files

Additional file 1: Database Search Strategy. (DOCX 15 kb)

Additional file 2: Quality Assessment of CASP cohort tool. (DOCX 31 kb)

Additional file 3: Study characteristics and results of selected publications. (DOCX 30 kb)

## Abbreviations

CASP: Critical Appraisal Skills Program
ERI: Effort reward imbalance model
FCM: Friction cost method
HCM: Human capital method
ICER: Incremental cost-effectiveness ratio
JD-C: Job-demands control model
JD-R: Job-demands resources model
MeSH: Medical subject headings
PAWs: Perceived ability to work scale
PRISMA: Preferred Reporting Items for Systematic Reviews and Meta-Analyses
SPS-6: Stanford presenteeism scale
WHO-HPQ: World Health Organization health and performance questionnaire
WLQ: Work limitations questionnaire
WPAI: Work productivity and activity impairment scale

## References

[1] Rainbow JG, Steege LM (2017). Presenteeism in nursing: an evolutionary concept analysis. Nurs Outlook.

[2] Quazi H. Presenteeism: the invisible cost to organizations. London: Palgrave Macmillan UK; 2013.

[3] Vroome E. Prevalence of sickness absence and'presenteeism'. European Foundation for the Improvement of living and working conditions (Eurofound); 2006.

[4] Heponiemi T, Kouvonen A, Sinervo T, Elovainio M (2013). Is the public healthcare sector a more strenuous working environment than the private sector for a physician?. Scandinavian journal of public health.

[5] Mandiracioglu A, Bolukbas O, Demirel M, Gumeli F (2015). Factors related to presenteeism among employees of the private sector. Int J Occup Saf Ergon.

[6] Dew K (2011). Pressure to work through periods of short term sickness. BMJ: British Medical Journal (Online).

[7] McKevitt C, Morgan M, Dundas R, Holland W (1997). Sickness absence and ‘working through’illness: a comparison of two professional groups. J Public Health.

[8] Hansen CD, Andersen JH (2008). Going ill to work--what personal circumstances, attitudes and work-related factors are associated with sickness presenteeism?. Soc Sci Med.

[9] Johansen V, Aronsson G, Marklund S (2014). Positive and negative reasons for sickness presenteeism in Norway and Sweden: a cross-sectional survey. BMJ Open.

[10] Aronsson G, Gustafsson K, Dallner M (2000). Sick but yet at work. An empirical study of sickness presenteeism. J Epidemiol Community Health.

[11] Cancelliere C, Cassidy JD, Ammendolia C, Côté P (2011). Are workplace health promotion programs effective at improving presenteeism in workers? A systematic review and best evidence synthesis of the literature. BMC Public Health.

[12] Goetzel RZ, Long SR, Ozminkowski RJ, Hawkins K, Shaohung W, Lynch W (2004). Health, absence, disability, and Presenteeism cost estimates of certain physical and mental health conditions Affectig U.S. employers. J. Occup. Environ. Med..

[13] Loeppke R, Taitel M, Haufle V, Parry T, Kessler RC, Jinnett K (2009). Health and productivity as a business strategy: a multiemployer study. J Occup Environ Med.

[14] Kirkham HS, Clark BL, Bolas CA, Lewis GH, Jackson AS, Fisher D (2015). Which modifiable health risks are associated with changes in productivity costs?. Popul. Health Manag..

[15] Caverley N, Cunningham JB, MacGregor JN (2007). Sickness presenteeism, sickness absenteeism, and health following restructuring in a public service organization. J Manag Stud.

[16] Cooper C, Dewe P (2008). Well-being—absenteeism, presenteeism, costs and challenges. Occup Med.

[17] Schultz AB, Chen C-Y, Edington DW (2009). The cost and impact of health conditions on presenteeism to employers. PharmacoEconomics.

[18] Letvak SA, Ruhm CJ, Gupta SN (2012). Nurses' presenteeism and its effects on self-reported quality of care and costs. AJN. Am J Nurs.

[19] Kocher R, Sahni NR (2011). Rethinking health care labor. N Engl J Med.

[20] Simpson R (1998). Presenteeism, power and organizational change: Long hours as a career barrier and the impact on the working lives of women managers. Br J Manag.

[21] Johns G. Presenteeism: a short history and a cautionary tale. Contemporary occupational health psychology: global perspectives on research and Practice. Wiley; 2012;2:204–20.

[22] Critical Appraisal Skills Programme (CASP). Cohort Study and Economic Evaluation Checklist 2017.

[23] Demerouti E, Le Blanc PM, Bakker AB, Schaufeli WB, Hox J (2009). Present but sick: a three-wave study on job demands, presenteeism and burnout. Career Dev Int.

[24] Dellve L, Hadzibajramovic E, Ahlborg G (2011). Work attendance among healthcare workers: prevalence, incentives, and long-term consequences for health and performance. J Adv Nurs.

[25] Trinkoff AM, Le R, Geiger-Brown J, Lipscomb J, Lang G (2006). Longitudinal relationship of work hours, mandatory overtime, and on-call to musculoskeletal problems in nurses. Am J Ind Med.

[26] LaVela S, Goldstein B, Smith B, Weaver FM (2007). Working with symptoms of a respiratory infection: staff who care for high-risk individuals. Am J Infect Control.

[27] Martinez LF, Ferreira AI (2012). Sick at work: presenteeism among nurses in a Portuguese public hospital. Stress Health.

[28] d'Errico A, Viotti S, Baratti A, Mottura B, Barocelli AP, Tagna M (2013). Low back pain and associated presenteeism among hospital nursing staff. J Occup Health.

[29] Letvak S, Ruhm C, Gupta S (2013). Differences in health, productivity and quality of care in younger and older nurses. J Nurs Manag.

[30] Sendén MG, Schenck-Gustafsson K, Fridner A (2016). Gender differences in reasons for sickness Presenteeism-a study among GPs in a Swedish health care organization. Ann Occup Environ Med.

[31] Sendén GM, Lovseth LT, Schenck-Gustafsson K, Fridner A (2013). What makes physicians go to work while sick: a comparative study of sickness presenteeism in four European countries (HOUPE). Swiss Med Wkly.

[32] Thun S, Fridner A, Minucci D, Løvseth LT. Sickness present with signs of burnout: the relationship between burnout and sickness presenteeism among university hospital physicians in four European countries. Scandinavian Psychologist. 2014;1(5). http://psykologisk.no/sp/2014/11/e5/.

[33] Boumans NP, Dorant E (2014). Double-duty caregivers: healthcare professionals juggling employment and informal caregiving. A survey on personal health and work experiences. J Adv Nurs.

[34] Umann J, Guido LD, Silva RM (2014). Stress, coping and presenteeism in nurses assisting critical and potentially critical patients. Revista da Escola de Enfermagem da USP.

[35] Yang T, Guo Y, Ma M, Li Y, Tian H, Deng J (2017). Job stress and Presenteeism among Chinese healthcare workers: the mediating effects of affective commitment. Int J Environ Res Public Health.

[36] Vandenbroeck S, Van Gerven E, De Witte H, Vanhaecht K, Godderis L (2017). Burnout in Belgian physicians and nurses. Occup Med.

[37] Warren CL, White-Means SI, Wicks MN, Chang CF, Gourley D, Rice M (2011). Cost burden of the presenteeism health outcome: diverse workforce of nurses and pharmacists. J Occup Environ Med.

[38] Aysun K, Bayram Ş (2017). Determining the level and cost of sickness presenteeism among hospital staff in Turkey. Int J Occup Saf Ergon.

[39] Rantanen I, Tuominen R (2011). Relative magnitude of presenteeism and absenteeism and work-related factors affecting them among health care professionals. Int Arch Occup Environ Health.

[40] Christensen JR, Kongstad MB, Sjøgaard G, Søgaard K (2015). Sickness presenteeism among health care workers and the effect of BMI, cardiorespiratory fitness, and muscle strength. J. Occup. Environ. Med..

[41] Noben C, Smit F, Nieuwenhuijsen K, Ketelaar S, Gärtner F, Boon B (2014). Comparative cost-effectiveness of two interventions to promote work functioning by targeting mental health complaints among nurses: pragmatic cluster randomised trial. Int J Nurs Stud.

[42] Noben C, Evers S, Nieuwenhuijsen K, Ketelaar S, Gartner F, Sluiter J (2015). Protecting and promoting mental health of nurses in the hospital setting: is it cost-effective from an employer's perspective?. Int J Occup Med Environ Health.

[43] Skela-Savič B, Pesjak K, Hvalič-Touzery S (2017). Low back pain among nurses in Slovenian hospitals: cross-sectional study. Int Nurs Rev.

[44] Johns G (2010). Presenteeism in the workplace: a review and research agenda. J Organ Behav.

[45] McIver JP, Carmines EG. Unidimensional scaling: Sage; 1981.

[46] Loftland JH, Pizzi L, Frick KD. A review of health-related workplace productivity loss instruments. Pharmacoeconomics. 2004;22(3):165–84. 10.2165/00019053-200422030-00003 14871164

[47] Stewart WF, Ricci JA, Leotta C (2004). Health-related lost productive time (LPT): recall interval and bias in LPT estimates. J Occup Environ Med.

[48] Zhang W, Bansback N, Anis AH (2011). Measuring and valuing productivity loss due to poor health: a critical review. Soc Sci Med.

[49] Gardner BT, Dale AM, Buckner-Petty S, Van Dillen L, Amick IIIBC, Evanoff B (2016). Comparison of employer productivity metrics to lost productivity estimated by commonly used questionnaires. Journal of occupational and environmental medicine/American college of. Occup Environ Med.

[50] Mattke S, Balakrishnan A, Bergamo G, Newberry SJ (2007). A review of methods to measure health-related productivity loss. Am J Manag Care.

[51] Reilly M, Bracco A, Ricci JF, Santoro J, Stevens T (2004). The validity and accuracy of the work productivity and activity impairment questionnaire–irritable bowel syndrome version (WPAI: IBS). Aliment Pharmacol Ther.

[52] Wang PS, Beck AL, Berglund P, McKenas DK, Pronk NP, Simon GE (2004). Effects of major depression on moment-in-time work performance. Am J Psychiatr.

[53] Schultz AB, Edington DW (2007). Employee health and presenteeism: a systematic review. J Occup Rehabil.

[54] Rice DP, Kelman S, Miller LS, Dunmeyer S. The economic costs of alcohol and drug abuse and mental illness, 1985. National Institute on Drug Abuse. 1990.

[55] Bakker AB, Demerouti E (2007). The job demands-resources model: state of the art. J Manag Psychol.

[56] Schaufeli WB, Taris TW. A critical review of the job demands-resources model: implications for improving work and health. In: Bridging occupational, organizational and public health: Springer; 2014. p. 43–68.

[57] Nielsen K, Daniels K (2016). The relationship between transformational leadership and follower sickness absence: the role of presenteeism. Work & Stress.

[58] Jacobs R, Mannion R, Davies HT, Harrison S, Konteh F, Walshe K (2013). The relationship between organizational culture and performance in acute hospitals. Soc Sci Med.

[59] Bakker AB, Demerouti E. Multiple levels in job demands-resources theory: implications for employee well-being and performance. Handbook of well-being. 2018.

[60] Lu L, Cooper CL, Yen Lin H. A cross-cultural examination of presenteeism and supervisory support. Career Dev Int. 2013;18(5):440–56.

