# Supplementary material for: Presenteeism exposures and outcomes amongst hospital doctors and nurses: a systematic review
Source: BMC Health Serv Res. 2018 Dec 19;18:985. doi: 10.1186/s12913-018-3789-z (PMC6299953; doi:10.1186/s12913-018-3789-z)
Supplement: Supplementary file 1 — Database Search Strategy. (DOCX 15 kb) [file 12913_2018_3789_MOESM1_ESM.docx]

**Additional File 1. Database Search Strategy**

| **Search Strategy** | #1 and #2 and #3 |
| --- | --- |
| **#1** | nurse* or physician* or doctor* or specialist* or “general practition*” or surgeon* or hospitalist* |
| **#2** | presenteeism or “work atten*” or “sick* presen*” or “sick* attend*” |
| **#3** | predictor*or consequence* or outcome* or cause* or antecedent* or “risk factor*” or associat* or correlat* or burnout or autonomy or “job demand*” or “job resource*” or stress or “job satisfaction” or turnover or “work tolerance” or “work schedule tolerance” or overtime or cost* or “financ* cost*” or outcome* or program* or evaluat* or intervention |
| **Filters** | Language: English  Date: Jan 1998-Dec 2017  Search for: Titles, Abstracts, All subject headings, keywords Article type: peer reviewed journal |
