# Supplementary material for: Presenteeism exposures and outcomes amongst hospital doctors and nurses: a systematic review
Source: BMC Health Serv Res. 2018 Dec 19;18:985. doi: 10.1186/s12913-018-3789-z (PMC6299953; doi:10.1186/s12913-018-3789-z)
Supplement: Supplementary file 2 — Quality Assessment of CASP cohort tool. (DOCX 31 kb) [file 12913_2018_3789_MOESM2_ESM.docx]

| **Additional File 2. Quality Assessment of CASP cohort tool** | | | | | | | | | | | | | | | | | | | | | | | | | | | | | | | | | | | | | | | | | | | | | | | |
| --- | --- | --- | --- | --- | --- | --- | --- | --- | --- | --- | --- | --- | --- | --- | --- | --- | --- | --- | --- | --- | --- | --- | --- | --- | --- | --- | --- | --- | --- | --- | --- | --- | --- | --- | --- | --- | --- | --- | --- | --- | --- | --- | --- | --- | --- | --- | --- |
|  | | | | 1 | 2 | 3 | 4 | | 5 | 6 | | 7 | 8 | 9 | 10 | 11 | 12 | | 13 | 14 | | 15 | 16 | 17 | 18 | 19 | 20 | | 21 | 22 | | 23 | 24 | 25 | 26 | 27 | 28 | | 29 | 30 | | 31 | 32 | 33 | 34 | 35 | 36 |
| 1. Did the study address a clearly focused issue? | | | | 1 | 1 | 1 | 1 | | 1 | 1 | | 1 | 1 | 1 | 1 | 1 | 1 | | 1 | 1 | | 1 | 1 | 1 | 1 | 1 | 1 | | 1 | 1 | | 1 | 1 | 1 | 1 | 1 | 1 | | 1 | 1 | | 1 | 1 | 1 | 1 | 1 | 1 |
| 2. Was the sample recruited in an acceptable way? | | | | 1 | 1 | 1 | 1 | | 1 | 1 | | 1 | 0 | 0 | 1 | 1 | 1 | | 1 | 0 | | 1 | 0 | 0 | 0 | 0 | 0 | | 0 | 1 | | 0 | 0 | 1 | 0 | 1 | 0 | | 0 | 1 | | 1 | 1 | 0 | 1 | 1 | 0 |
| 3. Was the exposure accurately measured to minimise bias? | | | | 1 | 1 | 1 | 0 | | 0 | 0 | | 0 | 1 | 1 | 1 | 1 | 1 | | 0 | 1 | | 1 | 1 | 1 | 1 | 1 | 1 | | 1 | 0 | | 0 | 0 | 0 | 0 | 1 | 1 | | 0 | 1 | | 1 | 1 | 1 | 0 | 1 | 1 |
| 4. Was the outcome accurately measured to minimise bias? | | | | 0 | 1 | 1 | 0 | | 0 | 0 | | 1 | 1 | 1 | 1 | 0 | 1 | | 1 | 0 | | 1 | 1 | 1 | 1 | 1 | 0 | | 1 | 0 | | 0 | 0 | 0 | 0 | 1 | 0 | | 0 | 1 | | 0 | 1 | 1 | 0 | 1 | 1 |
| 5a) Have the authors identified all important confounding factors? | | | | 0 | 0 | 0 | 0 | | 1 | 0 | | 1 | 0 | 0 | 0 | 1 | 0 | | 1 | 1 | | 0 | 0 | 0 | 0 | 0 | 0 | | 0 | 0 | | 1 | 0 | 0 | 0 | 0 | 0 | | 0 | 0 | | 0 | 0 | 0 | 1 | 0 | 0 |
| 5b) Have they taken account of the confounding factors in the design and/or analysis? | | | | 1 | 1 | 1 | 1 | | 1 | 0 | | 1 | 0 | 1 | 0 | 1 | 1 | | 1 | 1 | | 1 | 1 | 0 | 1 | 1 | 1 | | 0 | 0 | | 1 | 0 | 0 | 1 | 0 | 1 | | 1 | 1 | | 0 | 1 | 0 | 1 | 1 | 1 |
| 6a) Was the follow up of subjects complete enough? | | | | 0 | 1 | 1 | - | | - | - | | - | - | - | - | - | - | | - | - | | - | - | - | - | - | - | | - | - | | - | - | - | - | - | - | | - | - | | - | - | - | - | - | 0 |
| 6b) Was the follow up of subjects long enough? | | | | 1 | 1 | 1 | - | | - | - | | - | - | - | - | - | - | | - | - | | - | - | - | - | - | - | | - | - | | - | - | - | - | - | - | | - | - | | - | - | - | - | - | 1 |
| 7. What are the results of this study? | | | | - | - | - | - | | - | - | | - | - | - | - | - | - | | - | - | | - | - | - | - | - | - | | - | - | | - | - | - | - | - | - | | - | - | | - | - | - | - | - | - |
| 8. How precise are the results? | | | | 0 | 1 | 1 | 0 | | 1 | 0 | | 0 | 1 | 1 | 0 | 1 | 1 | | 0 | 0 | | 0 | 0 | 0 | 0 | 0 | 0 | | 0 | 0 | | 1 | 0 | 0 | 0 | 1 | 0 | | 1 | 0 | | 0 | 0 | 0 | 0 | 0 | 0 |
| 9. Do you believe the results? | | | | 1 | 1 | 1 | 1 | | 1 | 1 | | 1 | 0 | 0 | 0 | 1 | 1 | | 1 | 0 | | 1 | 0 | 0 | 0 | 0 | 0 | | 0 | 1 | | 1 | 0 | 0 | 0 | 1 | 1 | | 0 | 1 | | 1 | 1 | 0 | 1 | 0 | 0 |
| 10. Can the results be applied to the local population? | | | | 1 | 0 | 0 | 1 | | 0 | 0 | | 0 | 0 | 0 | 0 | 0 | 0 | | 0 | 0 | | 0 | 0 | 0 | 0 | 0 | 0 | | 0 | 0 | | 0 | 0 | 0 | 0 | 1 | 0 | | 0 | 1 | | 0 | 0 | 0 | 0 | 0 | 0 |
| 11. Do the results of this study fit with other available evidence? | | | | 1 | 1 | 1 | 1 | | 0 | 1 | | 1 | 0 | 0 | 1 | 1 | 1 | | 1 | 1 | | 1 | 0 | 0 | 0 | 0 | 1 | | 0 | 1 | | 1 | 1 | 1 | 0 | 1 | 1 | | 1 | 1 | | 1 | 1 | 0 | 1 | 1 | 1 |
| 12. What are the implications of this study for practice? | | | | - | - | - | - | | - | - | | - | - | - | - | - | - | | - | - | | - | - | - | - | - | - | | - | - | | - | - | - | - | - | - | | - | - | | - | - | - | - | - | - |
| **Total (12 for prospective, 10 for cross-sectional)** | | | | 8 | 10 | 10 | 6 | | 6 | 4 | | 7 | 4 | 5 | 5 | 8 | 8 | | 7 | 5 | | 7 | 4 | 3 | 4 | 4 | 4 | | 3 | 4 | | 6 | 2 | 3 | 2 | 8 | 5 | | 4 | 8 | | 5 | 7 | 3 | 6 | 6 | 6 |
| 1 | Demerouti 2009 | 8 | Umann2012 | | | | | 15 | | | Umann 2014 | | | | | | | 22 | | | Scyzmak2016 | | | | | | | 29 | | | Rostad 2017 | | | | | | | 36 | | | Christensen 2015 | | | | | | |
| 2 | Dellve 2011 | 9 | d'Errico2013 | | | | | 16 | | | Brborovic2014 | | | | | | | 23 | | | Senden2016 | | | | | | | 30 | | | Yang 2017 | | | | | | | 37 | | | Noben2014 | | | | | | |
| 3 | Trinkoff2006 | 10 | Letvak 2013 | | | | | 17 | | | Brborovic2016 | | | | | | | 24 | | | Al Nuhait 2017 | | | | | | | 31 | | | Aysun2017 | | | | | | | 38 | | | Noben2015 | | | | | | |
| 4 | Mckevit1997 | 11 | Heponiemi2013 | | | | | 18 | | | Brborovic2017 | | | | | | | 25 | | | Chiu2017 | | | | | | | 32 | | | Warren 2011 | | | | | | |  | | |  | | | | | | |
| 5 | LaVela 2007 | 12 | Sendén2013 | | | | | 19 | | | Pit2014 | | | | | | | 26 | | | Mossad2017 | | | | | | | 33 | | | Queiroz-Lima 2015 | | | | | | |  | | |  | | | | | | |
| 6 | Bracewell2010 | 13 | Thun2014 | | | | | 20 | | | Pit 2016 | | | | | | | 27 | | | Skela-Savič2017 | | | | | | | 34 | | | Rantanen2011 | | | | | | |  | | |  | | | | | | |
| 7 | Martinez 2012 | 14 | Boumans2014 | | | | | 21 | | | Pranckeviciene2016 | | | | | | | 28 | | | Vandenbroeck2017 | | | | | | | 35 | | | Letvak 2012 | | | | | | |  | | |  | | | | | | |

| **Quality Assessment - CASP Economic Evaluation tool** | | |
| --- | --- | --- |
|  | **37** | **38** |
| 1. Was a well-defined question posed? | 1 | 1 |
| 2. Was a comprehensive description of the competing alternatives given? | 1 | 1 |
| 3. Does the paper provide evidence that the programme would be effective ? | 1 | 1 |
| 4. Were the effects of the intervention identified | 1 | 1 |
| 5. Were all important and relevant resources required and health outcome costs for each alternative identified, measured in appropriate units and valued credibly? | 1 | 1 |
| 6. Were costs and consequences adjusted for different times at which they occurred (discounting)? | 0 | 0 |
| 7. What were the results of the evaluation? | - | - |
| 8. Was an incremental analysis of the consequences and cost of alternatives performed? | 1 | 1 |
| 9. Was an adequate sensitivity analysis performed? | 1 | 1 |
| 10. Is the programme likely to be equally effective in your context or setting? | 1 | 1 |
| 11. Are the costs translatable to your setting? | 0 | 0 |
| 12. Is it worth doing in your setting? | 1 | 1 |
| Total:11 marks | 9 | 9 |
