# Supplementary material for: Presenteeism exposures and outcomes amongst hospital doctors and nurses: a systematic review
Source: BMC Health Serv Res. 2018 Dec 19;18:985. doi: 10.1186/s12913-018-3789-z (PMC6299953; doi:10.1186/s12913-018-3789-z)
Supplement: Supplementary file 3 — Study characteristics and results of selected publications. (DOCX 30 kb) [file 12913_2018_3789_MOESM3_ESM.docx]

**Additional file 3. Study characteristics and results of selected publications**

| **Presenteeism exposures and outcomes studies** | | | |
| --- | --- | --- | --- |
| **Cohort studies** | | | |
| **Publications** | **Survey method /response rate** | **Statistical analysis** | **Results** |
| **Demerouti 2009**  Netherlands | *Paper (at work with postage)*  Nurses (N=258)  rr: T1 (74%), T2 (63%), T3 (53%) | Correlational analysis  Structural Equation Modelling | 50% nurses had worked sick at each timepoint. T2 Job demands led to T3 presenteeism (r=0.59). Presenteeism caused depersonalization, while exhaustion was recurrent with presenteeism over time. |
| **Dellve**  **2011**  Sweden | *Paper (mail to home)*  Non-leading Healthcare workers (N= 5300)  rr: T1 (61%), T2 (85%) | Crude prevalence ratios (PRs)  Cox regression for  adjusted RR | SP lower for doctors (47%) and nurses (48%) as to graduated professionals (53%). Attendance requirements and incentives were positively and negatively associated with SP respectively. SP increased risk of poor health and burnout in follow up. SP predicted current (OR=2.39, 95%CI:1.57–3.64) and long-term sick leave (OR=2.16 95%CI: 1.40–3.34), poor work ability (OR=2.31, 95%CI: 1.66–3.20) and lower performance (OR =1.82, 95%CI:1.11–3.00) at follow-up. BA reduced all above risks. |
| **Trinkoff 2006**  United States | *Paper (by mail)*  Nurses (N= 5000)  rr: T1(62%), T2 (85%), T3 (86%) | Principal components analysis  Logistic Regression | Working when supposed to be time off (work while sick, on a day off or vacation day, breaks taken) had higher risk in having neck (OR=1.32, 95% CI 1.06-1.64) , shoulder (OR =1.23, 95%CI: 1.01-1.50) and back (OR=1.12, 95%CI: 0.93-1.35) pain . |
| **Cross-sectional studies** | | | |
| **Mckevit 1997**  United Kingdom | *Paper (by mail)*  Hospital doctors  (N=669), general practitioners (GPs) (N=670), white collars (N=400)  rr: 74% | Chi-square tests  Logistic regression  Qualitative interviews | 86% GPs, 85% hospital doctors and 88% white-collars had SP. GPs (OR=4.1, 95% CI:1.2-2.4) and hospital doctors (OR= 2.7, 95%CI:1.9-3.7) were more likely than white collars not taking sick leave last year, doctors have higher risk of long sick leave (>7 days). Top presenteeism reasons were workload, job commitment, peer pressure, burdening colleagues, no substitution. |
| **LaVela**  **2007**  United States | *Paper (at work with postage)*  Health care workers (N=1552)  rr: 53% | Logistic regression | 86% worked sick with respiratory illness, those with institute having droplet control precautions (OR 0.42, P = 0.034), restricting inter-ward staff movement (OR 0.26, p=0.002), and inter-patient contact (OR 0.32, p= 0.01) less likely had SP. |
| **Martinez 2012**  Portugal | *Not specified*  Nurses (N=600)  rr: 49.3% | Correlational analysis | Top presenteeism conditions were lower-back pain, respiratory illness and migraines. Females had higher presenteeism, but no gender differences in duration. Older age had longer hours of presenteeism (r=0.167, p<0.05). Perceived health status less affected by SP (r = 0.175, p<0.01). Higher paid (r=0.146, p<0.05) and senior nurses (r=0.123, p<0.05) had less presenteeism. |
| **d'Errico 2013**  Italy | *Not specified*  Nurses (N=174)  rr: 91% | Univariate analysis  Poisson multivariable regression | Lower back pain (LBP) SP prevalence was 58.2%. Job title associated with SP (RR=0.42, p=0.001). Decreased SP risk when stooping often for long time (RR=0.63, p=0.002), most likely due to SA. Effects of department seniority, mental symptoms and LBP interference with daily activities on SP disappeared after adjustment. |
| **Letvak**  **2013**  United States | *Paper (mailed with postage, 3-weeks follow up postcard)*  Registered nurses (N=1256) rr: 47% | Two-sample t-test | Older nurses had higher health productivity loss of 0.20 (p=0.055) than younger nurses. |
| *Note: SP – sickness presenteeism, SA- sickness absence, rr- response rate, RR- risk ratio, OR – odds ratio, 95% CI – 95% confidence interval, α- Cronbach’s Alpha* | | | |

| **Presenteeism exposures and outcomes studies** | | | | | | |
| --- | --- | --- | --- | --- | --- | --- |
| **Cross-sectional studies** | | | | | | |
| **Publications** | **Survey method /response rate** | | **Statistical analysis** | | **Results** | |
| **Heponiemi 2013**  Finland | *Paper (by mail, follow up non-respondents twice)*  Physicians (N=5000)  rr: 57% | | Logistic/ linear regression | | 68% experienced SP. Private physicians had lower levels of SP (OR =0.64, 95% CI: 0.49–1.00), and those of 40-50 years old (OR= 0.95 (0.73–1.22). | |
| **Sendén**  **2013**  4 European countries | *Iceland: web, Norway, Sweden: web and paper, Italy: paper*  Physicians  Sweden (N=1031), Norway (N=354), Iceland (N =242), Italy (N=369) rr:53% | | Chi-square test,  ANOVA, correlational analysis, hierarchical multiple linear regression | | SP were 86% in Italy, 70% in Sweden, 76% in Norway and 75% in Iceland. SP increased with being in academia, sickness behaviour (taking compensatory leave, self-diagnosis and treatment), and decreased with organizational care. These variables explained 18.3%, 7.1%, 14.5% and 13.4% variance of SP in Sweden, Norway, Iceland and Italy respectively. Gender (female) was only associated with SP in Sweden. | |
| **Thun**  **2014**  4 European countries | *web and paper, Italy: paper only*  Physicians  Norway (n=378), Sweden (n=1074), Iceland (n=254), Italy (n=372) rr:52.6% | | Correlational analysis, hierarchical regression, one-way ANOVA | | SP was associated with disengagement (β = 0.07, p < .001) (21% variance) and exhaustion (β = 0.19, p < .001) ( 24% variance) when age, gender, country, superior and colleague support, control over work pace and decision making were included. | |
| **Boumans**  **2014**  Netherlands | *Web-based*  Healthcare workers (90.2% nurses) (N= 1573) rr:28.3% | | Chi-square test, linearity test, ANOVA,  ANCOVA | | Intensity of double care-givers were associated with presenteeism (0. 23, p<0.01) | |
| **Umann**  **2014**  Brazil | *Not specified*  Nurses (N=147)  rr: 88% | | Kolmogorov-Smirnov test, Chi-square test, correlational analysis | | 75% nurses had productivity loss of 4.84%, Productivity loss differ by department (pediatrics highest – 6.43%) , but no difference between units. Stress and productivity loss are associated with nurses caring for critically ill patients. | |
| **Senden**  **2016**  Sweden | *Web-based*  General practitioners  (N= 698)  rr: 41% | | univariate ANOVA,  Mediation analysis,  MANOVA | | 74 % women and 59 % men “sometimes/ often” work sick. Work-life conflict mediates gender on SP. Gender no longer predicted SP when work-life conflict was added. Gender had main effect on SP reasons when adjusted for health. (F(5, 236) = 5.076,p < 0.01) | |
| **Skela-Savič 2017**  Slovenia | *Not specified*  Nurses (n= 2777)  rr: 62.2% | | Correlational analysis, logistic regression,  Exploratory factor analysis | | Presenteeism (high SPS score =better work ability despite health problems) correlated with work dissatisfaction (Z =-8.528, p<0.001), limited lifting equipment (Z =-4.806, p<0.001), not a manager (Z =-2.805, p=0.005), lowered work activity (Z =-12.454, p<0.001) and physical activity (Z =-9.852, p<0.001), healthcare provision need (Z = -7.381, p<0.001) and LBP (Z =-4.229, p<0.001). | |
| **Yang**  **2017**  **China** | *Not specified*  Doctors (30.5%) nurses (42.3%) and others (N=1392)  rr: 91% | | Structural equation modelling, Mediation analysis with Sobel test | | Affective commitment (β=-0.27; p <0.001) correlated with presenteeism. Hindrance stress associated with affective commitment (β =-0.40, p <0.001) and presenteeism (β =0.26, p <0.001). Affective commitment mediated hindrance stress & presenteeism. | |
| **Vandenbroeck**  **2017**  Belgium | *Web-based (intranet/ email). 2-week email follow up)*  Physicians(N=1169), nurses (N=4531) rr: 26% | | Confirmatory Factor Analysis, Structural Equation Modelling | | Emotional exhaustion is positively associated to presenteeism (β=0.4, p<0.01), depersonalization is not significantly associated with presenteeism | |
| *Note: SP – sickness presenteeism, SA- sickness absence, rr- response rate, RR- risk ratio, OR – odds ratio, 95% CI – 95% confidence interval, α- Cronbach’s Alpha* | | | | | | |
| **Presenteeism financial costing studies** | | | | | |  |
| **Publications** | **Survey method /response rate** | **Costing method** | | **Results** | |  |
| **Warren**  **2011**  United States | *Web-based*  Nurses (N=112) and pharmacists (N=114)  rr: 85.9% | Human Capital Method | | Indirect cost was 12,605/professional and $700 million state-wide. Presenteeism prevalence was 52.65% with mean productivity decrease of 13.2%. Physical symptoms and mental conditions were positively associated with presenteeism. Top physical conditions associated with presenteeism were back and neck pain, allergies, sleep problems, chronic fatigue. Depression was most prevalent and had strongest association with presenteeism. Sickness under-treatment not associated with presenteeism, but never medically treated or completed pharmacotherapy was associated with less presenteeism. | |  |
| **Letvak**  **2012**  United States | See Letvak 2013 | Human Capital Method | | 62% had experienced presenteeism. Presenteeism associated with pain and depression, lower quality-of-care scores, more patient falls and medication errors. Total productivity loss costs were $15,541 / nurse and $37.3 billion nation-wide. | |  |
| **Aysun**  **2017**  Turkey | *Not specified*  Physicians (N=59), nurses (N=243) and others (N=1684)  rr: 59.9% | Human Capital Method | | 17,669 hours/TRY 281,690.95 lost during 2-weeks for all health problems, and productivity loss 19.92 hours/TRY 315.57 per staff. 86.96% healthcare workers had lost time due to health problems in past 2 weeks. The estimated financial burden per staff was TRY 7573.68 per year. SP highest among women, younger (30-39) age, nurses/midwife, and bad health (p<0.05). | |  |
| **Rantanen 2011**  Finland | *Paper (prepaid postage)*  Nurses (N=212) rr: 64.6%  physicians (N=60) rr: 53.3% | Contingent valuation method | | SP and SA costs were 273.75 euros/ person and 373.87 euros/person last month. 37.4% had SP, mean time working sick was 16 hours, with average productivity loss of 45.4%.  Job satisfaction (β= -0.039, p<0.001) and acute diseases (β= 2.971, p<0.001) was associated with SP. Less shift work was associated with absenteeism (β=-1.645, p=0.014) but not SP. | |  |
| *Note: SP – sickness presenteeism, SA- sickness absence, rr- response rate, RR- risk ratio, OR – odds ratio, 95% CI – 95% confidence interval, α- Cronbach’s Alpha* | | | | | |  |

| **Intervention studies - randomized control trials** | | | | |
| --- | --- | --- | --- | --- |
| **Publication** | **Survey method /response rate** | **Intervention** | **Statistical analysis** | **Results** |
| **Christensen 2015**  Denmark | *Not specified*  *Surveys filled in after physical measurement tests*  Female health care workers (N=139)  rr: T1(68.8%),  T2 (61.9%),  T3 (55.0%) | Diet, physical exercise, and cognitive behavioral training.  0–3 months  dietary change advice, calorie counting, weight loss targets, cognitive behavioral training, strengthening exercises, and leisure time physical activity.  3–12 months  weight loss, physical exercise and cognitive behavioral training | Partial correlational analysis,  one-way analysis of variance with Tukey HSD posthoc  testing | SP associated with low total maximal voluntary contraction (MVC) (baseline: r= 0.312, p=0.023; 3-months: r=0.373, p=0.046) and high BMI (baseline: r=0.221, p=0.024; 3-months: r=0.326, p=0.004). Cardiorespiratory fitness (CRF) was not associated with job performance. No change in performance for 2nd round intervention (3-12 months), maybe due to participants not recalling improved functionality. |

| **Economic evaluation studies** | | | | | |
| --- | --- | --- | --- | --- | --- |
| **Publications** | **Survey method /response rate** | **Intervention Imposed** | **Costing method** | **Statistical analysis** | **Results** |
| **Noben**  **2014**  Netherlands | *Not specified*  Nurses under  occupational physician treatment (n=207)  e-mental treatment (n=204)  control (n=206) | 3 types of treatment after screening:  - Feedback and referral to occupational physician follow-up  - Feedback and referral and access to preventive e-mental health interventions  - Control – no feedback and referral to either intervention | Human capital method | **Cost-effectiveness analysis** (societal perspective - costs and benefits irrespective of cost bearer/ receiver)  *Incremental cost-effectiveness ratio (ICER)* (net costs/ savings per treatment responder)  *Stochastic uncertainty* - non-parametric bootstraps and cost-effectiveness 4-quandrant planes | The occupational physician intervention (ICER: 0.033) was recommended over e-mental health intervention (ICER:-0.047) after web mental health screening.  Total incremental costs for intervention was 64 euros/nurse. Total incremental benefits were 308 euros/nurse for absenteeism and 407 euros/nurse. |
| **Noben**  **2015**  Netherlands | *Not specified*  Nurses in an academic hospital  Final control arm (n=211) and experimental arm (n=210) | Control: screening without feedback and unrestricted access to usual care (N = 206)  Experimental: feedback and referral to the occupational physician for screened positive nurses (N = 207). | Human capital method | **Cost Benefit Analysis**  *The net benefits*  *Cost-to-benefit ratio*  *Return on investment*  *Stochastic uncertainty* - non-parametric bootstraps | Net-savings of 244 euros/nurse on absenteeism only, and 651 euros when absenteeism and presenteeism are considered. 5 euros up to 11 euros for every euro invested. Cost is covered after running the program for half a year |
